# Effects of Diet-Induced Obesity and Deficient in Vitamin D on Spermatozoa Function and DNA Integrity in Sprague-Dawley Rats

**DOI:** 10.1155/2018/5479057

**Published:** 2018-11-25

**Authors:** O. Merino, R. Sánchez, B. M. Gregorio, F. J. Sampaio, J. Risopatrón

**Affiliations:** ^1^Center of Biotechnology in Reproduction (CEBIOR-BIOREN), Universidad de La Frontera, Temuco, Chile; ^2^Center for Excellence in Morphological and Surgical Studies (CEMyQ), Universidad de La Frontera, Temuco, Chile; ^3^Doctoral Program in Morphological Sciences, Faculty of Medicine, Universidad de La Frontera, Temuco, Chile; ^4^Department Preclinical Sciences, Faculty of Medicine, Universidad de La Frontera, Temuco, Chile; ^5^Department of Anatomy State University of Rio de Janeiro, UERJ, Urogenital Research Unit, Biomedical Center, Rio de Janeiro, Brazil; ^6^Department of Basic Sciences, Faculty of Medicine, Universidad de La Frontera, Temuco, Chile

## Abstract

Obesity has adverse effects on male fertility and usually is diagnosed with a prevalence of vitamin D deficiency (VD^−^). Discussion on the impact of obesity/VD^−^ on sperm function has been limited. This study analyzed the effects of diet-induced obesity/VD^−^ on viability and plasma membrane integrity (PMI), superoxide anion (O_2_^−^) level, and DNA fragmentation (DNA_frag_) in sperm Sprague-Dawley rats. The males were randomized into four groups and fed for a period of 12 weeks: G1: control diet with vitamin D (C/VD^+^), G2: control diet without vitamin D (C/VD^−^), G3: high-fat diet with vitamin D (HF/VD^+^), and G4: high-fat diet without vitamin D (HF/VD^−^). Sperm function parameters were analyzed by flow cytometry. PMI percentages and O_2_^−^ levels were not affected by any of the diets. DNA fragmentation was increasing significantly (p<0.05) in the spermatozoa of animals with diets vitamin D deficient (G2) and diet-induced obesity (G4). Our results allow us to point out that diet-induced obesity and VD^−^ produce greater damage in DNA sperm of rats. The use of nutraceuticals containing vitamin D could be reducing the risk of fragmentation of DNA in spermatozoa.

## 1. Introduction

Obesity has adverse effects on male fertility and is an acknowledged risk factor for male subfertility [1, 2], this condition may result in hypogonadism, increased scrotal temperatures, impaired spermatogenesis, sperm decrease in the motility and concentration, alterations in morphology and mitochondrial function, and increased sperm DNA damage, affecting male reproductive health [3–7]. In animal and humans, a diet rich in fat or carbohydrates has been shown to contribute to the development of obesity and altered sperm parameters [8, 9].

A significant inverse correlation exists between obesity and vitamin D (VD) in males; however, there is uncertainty as to what the health consequences of these lower concentrations might be [10–14]. The action of VD is mediated by VD receptor (VDR) and enzymes that metabolize VD (CYP2R1, CYP27B1, and CYP24A1) that are highly expressed in testis, epididymis, seminal vesicle, prostate, and spermatozoa [15–24], suggesting a local regulation of active VD that may be important for spermatogenesis and/or sperm function.

The effects of diet-induced obesity/deficiency vitamin D (VD^−^) on sperm function have been limitedly discussed; the knowledge of the effects of this health condition on sperm quality would help understand the low fertility potential in obese/VD^−^ males. The aim of the study was to evaluate the effects of diet-induced obesity /VD^−^ on the PMI, O_2_^−^ and DNA_frag_ in Sprague-Dawley rats.

## 2. Material and Methods

### 2.1. Animals and Treatments

The experimental protocol study was approved by the Scientific Ethics Committee (CEC) of Universidad of La Frontera, Temuco, Chile (Protocol N° 167/15; Act N° 014/2016).

Twenty healthy male Sprague-Dawley rats (weight: 363±42.21 g, 4 months old) were used in this study. The animals were maintained in the Biotherius of Center for Excellence in Morphological and Surgical Studies (CEMyQ) of Universidad de La Frontera, Temuco, Chile; they were allowed free access to food (AIN-93M. PragSoluções Biociências®, Brasil) and water at all times and were maintained at a temperature of 21±2°C and controlled light cycle (12-12 h light/dark). Light in the room that housed the rats was provided by incandescent lighting, and all potential sources of ultraviolet light were eliminated to exclude the possibility of endogenous VD production in the skin [25]. The animal health status was periodically checked by a veterinarian.

After a period of acclimatization of four months, the animals were randomized into four groups (n=5 per group) according to the content high fat (HF) and vitamin D (VD) of their diets: G1: control diet with vitamin D (C/VD^+^), G2: control diet without vitamin D (C/VD^−^), G3: high-fat diet with vitamin D (HF/VD^+^), and G4: high-fat diet without vitamin D (HF/VD^−^).

The composition of nutrients in different diets was prepared following the recommendations of the AIN-93M [26] by PragSoluções Biociências® [27]. The groups received the diets over 12 weeks, from four months until seven months of age. The food intake was recorded daily. All body values were performed by our group and published by Merino et al. [27, 28].

### 2.2. Sperm Collection

The animals were euthanized according to the procedures described by Underwood* et al*. [29] prior to the sperm collection. Both epididymides, free of fat, were removed and placed in a polystyrene culture dish containing 5 mL gamete buffer medium (COOK®; Cook Medical Inc. Bloomington, Indiana, USA). Sperm samples were collected from the distal cauda epididymis by gentle massage and stripping expelling an approximately 3 cm stream of epididymal fluid, with special care to avoid blood [30, 31]. Semen samples were diluted in gamete buffer medium and adjusted to 4x10^6^ sperm/mL for sperm function evaluations.

### 2.3. Sperm Function Evaluations

#### 2.3.1. Sperm Viability and Plasma Membrane Integrity (PMI)

PMI was assessed using the LIVE/DEAD Sperm Viability kit (SYBR-14 dye/ PI; Invitrogen Inc., Eugene, OR, USA) according to Gravance* et al*. [30] with some modifications. Briefly, 250 *μ*L of sperm suspension + 2.5 *μ*L SYBR-14 (0.01 mmol, final concentration) + 1.25 *μ*L propidium iodide (PI; 0.01 mmol, final concentration) was incubated for 10 min at 37°C in the dark and immediately analyzed by flow cytometry. Spermatozoa positive to SYBR‐14 (SYBR‐14^+^) and negative to PI (PI^−^) were considered viable with intact plasma membrane. The analysis in each trial was replicated three times.

#### 2.3.2. Superoxide Anion Production (*O*_2_^−^)

The O_2_^−^ production was analyzed as previously described [32]. Briefly, 250 *μ*L of sperm suspension was incubated for 10 min at 37°C with 2 *μ*L (2 mmol) of dihydroethidium (DHE; Molecular Probes, Life Technologies) and in order to exclude dead cells from the analysis, 0.3 *μ*L (0.5 mmol) of SYTOX® Green was used (Molecular Probes, Life Technologies,) and analyzed by flow cytometry. The analysis in each trial was replicated three times. Spermatozoa positive to DHE (DHE^+^) and negative to SYTOX® Green (SYTOX® Green^−^) were considered with high O_2_^−^ production and viable.

#### 2.3.3. DNA Fragmentation (*DNA*_frag_)

DNA was evaluated according to Abbasihormozi* et al*. [33] with some modifications. Briefly, 250 *μ*L of sperm suspension was fixed in 4% formaldehyde for 10 min at 4°C and permeabilized in 100 *μ*L of 0.5% Triton X-100 (0.1% sodium citrate) for 15 min at 4°C. After washing in PBS, the pellet was resuspended in 50 *μ*L of TUNEL reaction mixture (Roche, Mannheim, Germany) and incubated for 60 min at 37°C in a humidified atmosphere in the dark. Next, 2 *μ*L of PI was added. Samples were immediately analyzed by flow cytometry. Spermatozoa stained green (TUNEL positive) were considered with fragmented DNA. Each sample was analyzed in triplicate.

#### 2.3.4. Flow Cytometry

A FACS Canto II™ flow cytometer (Becton Dickinson, Biosciences, San José, California, USA) using controlled by the software FACSDiva™ v. 6.1.3 was used to determine sperm viability and plasma membrane integrity (PMI: SYBR-14/PI), Superoxide anion level (O_2_^−^: DHE/SYTOX® Green), and DNA fragmentation (DNA_frag_: TUNEL), and 10 000 events were acquired by each test.

### 2.4. Statistical Analysis

The data were analyzed with the statistical program PRISM® version 6.0 (GraphPad Software, Inc., San Diego, CA, USA): differences of the sperm parameters, plasma membrane integrity, superoxide anion level, and DNA fragmentation. Statistical analysis of two-way ANOVA was used. Differences between groups were established using Tukey's multiple comparison tests. The effects of the diet-induced obesity and the vitamin D deficiency, as independent factors, and the possible interactions between both factors were tested by two-way ANOVA and Bonferroni's multiple comparisons test. The level of significance of p < 0.05 was established. Results are presented as mean ± SD.

## 3. Results

### 3.1. Glucose Concentrations

The glucose concentration (mmol/L) did not differ among the groups (P>0.05). Similarly, there were no differences in glucose concentrations before and after the experimental period in each group (G1:5.11/4.96, G2: 5.01/4.82, G3: 4.98/5.36, and G4: 4.84/5.15; P>0.05).

### 3.2. Sperm Function

#### 3.2.1. Viability and Plasma Membrane Integrity

**Figure 1:**
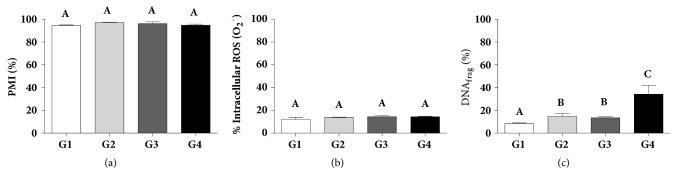
Effects of diet-induced obesity and vitamin D deficiency in sperm function in rats. (a) Sperm viability and membrane plasma integrity (PMI), (b) superoxide anion level (O_2_^−^), and (c) DNA fragmentation (DNA_frag_). G1: control diet with vitamin D (C/VD^+^), G2: control diet without vitamin D (C/VD^−^), G3: high-fat diet with vitamin D (HF/VD^+^), and G4: high-fat diet without vitamin D (HF/VD^−^). Data are expressed as mean ± SD and analysed by two-way ANOVA and Tukey's multiple comparisons test. Different superscript letters indicate significantly different rates (p<0.05, n=11).

**Figure 2:**
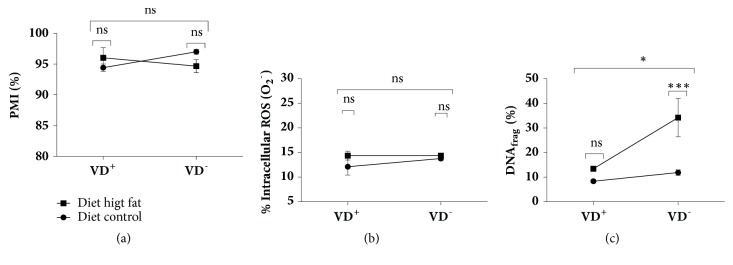
Interaction between diet-induced obesity and vitamin D on sperm function rat. (a) Interaction between diet-induced obesity and vitamin D on sperm viability and plasma membrane integrity (PMI), (b) interaction between diet-induced obesity and vitamin D on superoxide anion production (O_2_^−^), and (c) interaction between diet-induced obesity and vitamin D on DNA fragmentation (DNA_frag_). Data are expressed as mean ± SD and analysed by two-way ANOVA and Bonferroni's multiple comparisons test. Different asterisks indicate significantly different rates (p<0.05, n=11).

Sperm viability and plasma membrane integrity percentages were not affected by any of the diets (Figure 1(a)). The interaction between the variables deficiency of vitamin D and diet-induced obesity was not significant in sperm viability and plasma membrane integrity (F = 3.56; P-value = 0.0666). (Figure 2(a)).

#### 3.2.2. Superoxide Anion Production (*O*_2_^−^)

Sperm anion superoxide production was not affected by any of the diets (Figure 1(b)). The interaction between the variables deficiency of vitamin D and diet-induced obesity was not significant in the sperm anion superoxide production (F = 0.69; P-value = 0.4113) (Figure 2(b)).

#### 3.2.3. DNA Fragmentation (*DNA*_frag_)

DNA fragmentation increased significantly in the diets G2, G3 (14.6±2.5% and 13.4±3.2, resp.) and in G4 (34.21±7.8%), compared to the control (Figure 1(c)); besides, the interaction between the variables deficiency of vitamin D and diet-induced obesity was significant in DNA (F = 4.71; P-value = 0.0359) (Figure 2(c)).

## 4. Discussion

Obesity has adverse effects on male fertility and usually has been associated with a prevalence of VD^−^ [3–7, 10–14]; however, the effects of diet-induced obesity/VD^−^ on sperm function have been limited. The knowledge of the effects of this health condition on sperm quality would help understand the low fertility potential in obese/VD^−^ males. In this regard, in the present study, we evaluated the effects of diet-induced obesity/VD^−^ on the PMI, O_2_^−^ and DNA_frag_ in Sprague-Dawley rats.

The high-fat diet used in the present study was effective in promoting obesity, as demonstrated by significantly higher BM and the increase in adipose index. This condition was exacerbated by ingestion of high-fat diet and VD^−^ [27], without affecting serum glycemic profile. Glucose metabolism is an important event in spermatogenesis as well as specific functions, such as motility and fertilization ability in mature sperm [34].

The results revealed good quality of spermatozoa from control group sperm (G1) with high percentages of PMI intact, and low percentages of O_2_^−^ synthesis and DNA_frag_; nevertheless, the groups of males fed a diet-induced obesity and VD^−^ have impaired spermatozoa as evidenced by increased sperm DNA damage.

The PMI was not affected by diet-induced obesity. These results are in agreement with those previously reported in rabbits sperm [35]. In addition, it should be noted that PMI was not affected by concentrations of VD use in the diets. VD has an important role in sperm PMI [17]; the beneficial effect is dose-dependent at lower concentrations [17, 36, 37]; in the male gamete sperm it is necessary for its activation and adaptation to change its environment [17, 36].

ROS are highly reactive oxidizing agents that, at physiologic levels, are naturally involved in various physiologic pathways essential for normal reproduction; they are equilibrated by the presence of various enzymatic and nonenzymatic antioxidants that scavenge and neutralize excessive, and therefore detrimental amounts of ROS [38, 39]. The disturbance in the redox state causes oxidative stress (OS) which elicits its detrimental effects DNA damage [38, 39]. In rat sperm, O_2_^−^ and hydrogen peroxide (H_2_O_2_) have been used to determine intracellular (H_2_O_2_) or mitochondrial (O_2_) ROS in male diet-induced obesity [28], demonstrating that these increases cause DNA_frag_ sperm. Our results are in agreement with sperm DNA_frag_; however, in our study, low O_2_^−^ intracytoplasmic production was obtained. In addition, our results provide evidence that there exists an interaction between the variables (diet-induced obesity and VD^−^) with a significant effect on DNA status_,_ inducing fragmentation of DNA in epididymal sperm of rat. The interaction fat (obesity) by VD^−^ has been previously reported in rats and humans, about the vitamin D is stored in body fat, and despite an ostensibly adequate input, the obese male cannot readily access his or her own reserves of the vitamin dissolved in the fat of adipose tissue [10–14, 40–44] causing vitamin D deficiency in these males. On the other hand, previous studies have shown that vitamin D can prevent DNA damage directly or indirectly by inducing cell cycle arrest and increasing the activity of DNA repair [45]. This explains the interaction fat (obesity) by VD^−^ and the effect on DNA fragmentation in epididymal sperm of rat, observed in our study. To our knowledge, this is the first study that analyzes the interaction of obesity and VD- on sperm DNA. Possibly, the DNA damage observed could explain the low fertility potential in obese/VD^−^ males reported in previous studies [3–7, 10–14, 28].

The greatest influence of vitamin D on DNA could occur during spermatogenesis stage, in spermatogenesis [18, 46–48], because in this stage a marked expression of VDR and all the VD metabolizing enzymes has been evidenced [18]. The role of the VDR in sperm nucleus is unclear, suggesting that VDR in sperm nucleus could be implicated in a protective effect on DNA integrity [15, 23, 37, 45]. Nair-Shalliker et al. [45] suggest that DNA integrity in lymphocytes male VD^−^ may be more susceptible to damage when exposed to damaging agents.

Our results suggest a role of the VD as a protective genomic factor of sperm DNA integrity, in the stages of spermatogenesis; its deficiency significantly increased the DNA damage in male rats of normal weight and obesity [18, 21, 46, 49–53].

## 5. Conclusions

These findings suggest that appropriate vitamin D level in the diet is very important for sperm DNA integrity and consequently for rat reproduction. The use of nutraceuticals containing vitamin D could be important in the association of infertility and obesity in mammals.

## Data Availability

The data that supporting the conclusions generated of this study are included in this article.

## References

[1] Andersen J. M., Herning H., Aschim E. L. (2015). Body mass index is associated with impaired semen characteristics and reduced levels of anti-Müllerian hormone across a wide weight range. *PLoS ONE*.

[2] Bieniek J. M., Kashanian J. A., Deibert C. M. (2016). Influence of increasing body mass index on semen and reproductive hormonal parameters in a multi-institutional cohort of subfertile men. *Fertility and Sterility*.

[3] Kort H. I., Massey J. B., Elsner C. W. (2006). Impact of body mass index values on sperm quantity and quality. *Journal of Andrology*.

[4] Hammoud A. O., Wilde N., Gibson M., Parks A., Carrell D. T., Meikle A. W. (2008). Male obesity and alteration in sperm parameters. *Fertility and Sterility*.

[5] Kasturi S. S., Tannir J., Brannigan R. E. (2008). The metabolic syndrome and male infertility. *Journal of Andrology*.

[6] Chavarro J. E., Toth T. L., Wright D. L., Meeker J. D., Hauser R. (2010). Body mass index in relation to semen quality, sperm DNA integrity, and serum reproductive hormone levels among men attending an infertility clinic. *Fertility and Sterility*.

[7] Campbell J. M., Lane M., Owens J. A., Bakos H. W. (2015). Paternal obesity negatively affects male fertility and assisted reproduction outcomes: A systematic review and meta-analysis. *Reproductive BioMedicine Online*.

[8] Wilson G. T. (2010). Eating disorders, obesity and addiction. *European Eating Disorders Review*.

[9] Ferramosca A., Conte A., Damiano F., Siculella L., Zara V. (2014). Differential effects of high-carbohydrate and high-fat diets on hepatic lipogenesis in rats. *European Journal of Nutrition*.

[10] Wortsman J., Matsuoka L. Y., Chen T. C., Lu Z., Holick M. F. (2000). Decreased bioavailability of vitamin D in obesity. *The American journal of clinical nutrition*.

[11] Panidis D., Balaris C., Farmakiotis D. (2005). Serum parathyroid hormone concentrations are increased in women with polycystic ovary syndrome. *Clinical Chemistry*.

[12] Herranz S. A., García C. M. M., Alvarez V. D. F., García C. M. M. (2010). Vitamin D deficiency in morbidly obese patients. A case-control study. *Endocrinologia y nutricion: organo de la Sociedad Espanola de Endocrinologia y Nutricion*.

[13] Lorenzo J., Boente R., Sas Fojón M. (2012). Vitamin D deficiency and obesity. *Endocrinología y Nutrición*.

[14] Vanlint S. (2013). Vitamin D and obesity. *Nutrients*.

[15] Corbett S. T., Hill O., Nangia A. K. (2006). Vitamin D receptor found in human sperm. *Urology*.

[16] Nangia A. K., Hill O., Waterman M. D., Schwender C. E. B., Memoli V. (2007). Testicular Maturation Arrest to Testis Cancer: Spectrum of Expression of the Vitamin D Receptor and Vitamin D Treatment In Vitro. *The Journal of Urology*.

[17] Aquila S., Guido C., Perrotta I., Tripepi S., Nastro A., Andò S. (2008). Human sperm anatomy: Ultrastructural localization of 1*α*,25-dihydroxyvitamin D3 receptor and its possible role in the human male gamete. *Journal of Anatomy*.

[18] Blomberg Jensen M., Nielsen J. E., Jørgensen A. (2010). Vitamin D receptor and vitamin D metabolizing enzymes are expressed in the human male reproductive tract. *Human Reproduction*.

[19] Blomberg Jensen M., Bjerrum P. J., Jessen T. E. (2011). Vitamin D is positively associated with sperm motility and increases intracellular calcium in human spermatozoa. *Human Reproduction*.

[20] Luk J., Torrealday S., Neal Perry G., Pal L. (2012). Relevance of vitamin D in reproduction. *Human Reproduction*.

[21] Jensen M. B. (2014). Vitamin D and male reproduction. *Nature Reviews Endocrinology*.

[22] Jin H., Huang Y., Jin G. (2015). The vitamin D receptor localization and mRNA expression in ram testis and epididymis. *Animal Reproduction Science*.

[23] Mahmoudi A. R., Zarnani A. H., Jeddi-Tehrani M. (2013). Distribution of vitamin D receptor and 1*α*-hydroxylase in male mouse reproductive tract. *Reproductive Sciences*.

[24] Yao X., EI-Samahy M. A., Yang H. (2018). Age-associated expression of vitamin D receptor and vitamin D-metabolizing enzymes in the male reproductive tract and sperm of Hu sheep. *Animal Reproduction Science*.

[25] Kwiecinski G. G., Petrie G. I., DeLuca H. F. (1989). Vitamin D is necessary for reproductive functions of the male rat. *Journal of Nutrition*.

[26] Reeves P. G., Nielsen F. H., Fahey G. C. (1993). AIN-93 purified diets for laboratory rodents: final report of the American Institute of Nutrition ad hoc writing committee on the reformulation of the AIN-76A rodent diet. *Journal of Nutrition*.

[27] Merino O., Gregório B., Sampaio F., Sánchez R., Risopatrón J. (2017). Rol de la Vitamina D en el Desarrollo de la Obesidad. *International Journal of Morphology*.

[28] Merino O., Sánchez R., Gregorio M. B., Sampsaio F., Risopatrón J. (2018). Effect of high-fat and vitamin D deficient diet on rat sperm quality and fertility. *Theriogenology*.

[29] Underwood W., Anthony R., Gwaltney-Brant S., Poison A. S. P. C. A., Meyer R. (2013). *AVMA guidelines for the euthanasia of animals*.

[30] Gravance C. G., Garner D. L., Miller M. G., Berger T. (2001). Fluorescent probes and flow cytometry to assess rat sperm integrity and mitochondrial function. *Reproductive Toxicology*.

[31] Gravance C. G., Garner D. L., Miller M. G., Berger T. (2003). Flow cytometric assessment of changes in rat sperm mitochondrial function after treatment with pentachlorophenol. *Toxicology in Vitro*.

[32] De Iuliis G. N., Wingate J. K., Koppers A. J., McLaughlin E. A., Aitken R. J. (2006). Definitive evidence for the nonmitochondrial production of superoxide anion by human spermatozoa. *The Journal of Clinical Endocrinology & Metabolism*.

[33] Abbasihormozi S., Shahverdi A., Kouhkan A., Cheraghi J., Akhlaghi A. A., Kheimeh A. (2013). Relationship of leptin administration with production of reactive oxygen species, sperm DNA fragmentation, sperm parameters and hormone profile in the adult rat. *Archives of Gynecology and Obstetrics*.

[34] Ding G. L., Liu Y., Liu M. E. (2015). The effects of diabetes on male fertility and epigenetic regulation during spermatogenesis. *Asian Journal of Andrology*.

[35] Saez Lancellotti T. E., Boarelli P. V., Romero A. A. (2013). Semen Quality and Sperm Function Loss by Hypercholesterolemic Diet Was Recovered by Addition of Olive Oil to Diet in Rabbit. *PLoS ONE*.

[36] Aquila S., Guido C., Middea E. (2009). Human male gamete endocrinology: 1alpha, 25-dihydroxyvitamin D3 (1,25(OH)2D3) regulates different aspects of human sperm biology and metabolism. *Reproductive Biology and Endocrinology*.

[37] Zanatta L., Zamoner A., Zanatta A. P. (2011). Nongenomic and genomic effects of 1*α*,25(OH)2 vitamin D3 in rat testis. *Life Sciences*.

[38] Sharma R., Biedenharn K. R., Fedor J. M., Agarwal A. (2013). Lifestyle factors and reproductive health: Taking control of your fertility. *Reproductive Biology and Endocrinology*.

[39] Agarwal A., Majzoub A. (2017). Role of antioxidants in assisted reproductive techniques. *The World Journal of Men's Health*.

[40] Walsh J. S., Evans A. L., Bowles S. (2016). Free 25-hydroxyvitamin D is low in obesity, but there are no adverse associations with bone health. *American Journal of Clinical Nutrition*.

[41] Savastano S., Barrea L., Savanelli M. C. (2017). Low vitamin D status and obesity: Role of nutritionist. *Reviews in Endocrine and Metabolic Disorders*.

[42] Heaney R. P., Horst R. L., Cullen D. M., Armas L. A. G. (2009). Vitamin D3 Distribution and status in the body. *Journal of the American College of Nutrition*.

[43] Drincic A. T., Armas L. A. G., van Diest E. E., Heaney R. P. (2012). Volumetric dilution, rather than sequestration best explains the low vitamin D status of obesity. *Obesity*.

[44] Drincic A., Fuller E., Heaney R. P., Armas L. A. G. (2013). 25-Hydroxyvitamin D response to graded vitamin D3 supplementation among obese adults. *The Journal of Clinical Endocrinology & Metabolism*.

[45] Nair-Shalliker V., Fenech M., Forder P. M., Clements M. S., Armstrong B. K. (2012). Sunlight and vitamin D affect DNA damage, cell division and cell death in human lymphocytes: A cross-sectional study in South Australia. *Mutagenesis*.

[46] Schagdarsurengin U., Steger K. (2016). Epigenetics in male reproduction: Effect of paternal diet on sperm quality and offspring health. *Nature Reviews Urology*.

[47] McPhersson S. M. G., Longo F. J. (1993). Nicking of rat spermatid and spermatozoa DNA: Possible involvement of DNA topoisomerase II. *Developmental Biology*.

[48] Sakkas D., Alvarez J. G. (2010). Sperm DNA fragmentation: mechanisms of origin, impact on reproductive outcome, and analysis. *Fertility and Sterility*.

[49] Du Plessis S. S., Cabler S., McAlister D. A., Sabanegh E., Agarwal A. (2010). The effect of obesity on sperm disorders and male infertility. *Nature Reviews Urology*.

[50] Karlic H., Varga F. (2011). Impact of vitamin D metabolism on clinical epigenetics. *Clinical Epigenetics*.

[51] Pereira F., Barbáchano A., Singh P. K., Campbell M. J., Muñoz A., Larriba M. J. (2012). Vitamin D has wide regulatory effects on histone demethylase genes. *Cell Cycle*.

[52] Pike J. W., Meyer M. B., Bishop K. A. (2012). Regulation of target gene expression by the vitamin D receptor—an update on mechanisms. *Reviews in Endocrine and Metabolic Disorders*.

[53] Fetahu I. S., Höbaus J., Kállay E. (2014). Vitamin D and the epigenome. *Frontiers in Physiology*.

